# An easy method for preparation of Cre-loxP regulated fluorescent adenoviral expression vectors and its application for direct reprogramming into hepatocytes

**DOI:** 10.1016/j.btre.2016.10.003

**Published:** 2016-10-06

**Authors:** Chitose Kurihara, Koji Nakade, Jianzhi Pan, Jing Huang, Bohdan Wasylyk, Yuichi Obata

**Affiliations:** a Gene Engineering Division, RIKEN BioResource Center, 3-1-1 Koyadai, Tsukuba, Ibaraki 305-0074, Japan; b Institute of Animal Husbandry and Veterinary, Zhejiang Academy of Agricultural Sciences, 198, Shiqiao Rd., Hangzhou, Zhejiang, PR China; c Institut de Génétique et de Biologie Moléculaire et Cellulaire, 1, Rue Laurent Fries, Illkirch Cedex 67404, France

**Keywords:** Adenoviral vector, Cre-loxP, Fluorescent protein, Induced- hepatocyte, Direct reprogramming, Diffrentiation

## Abstract

The recombinant adenoviral gene expression system is a powerful tool for gene delivery. However, it is difficult to obtain high titers of infectious virus, principally due to the toxicity of the expressed gene which affects on virus replication in the host HEK293 cells. To avoid these problems, we generated a Cre-loxP-regulated fluorescent universal vector (termed pAxCALRL). This vector produces recombinant adenoviruses that express the red fluorescent protein (RFP) instead of the inserted gene during proliferation, which limits toxicity and can be used to monitor viral replication. Expression of the gene of interest is induced by co-infection with an adenovirus that expresses Cre-recombinase (AxCANCre). Recombinant adenovirus produced by this system that express Hnf4α and Foxa2 were used to reprogram mouse embryo fibroblast (MEF) into induced-hepatocyte-like cells (iHep) following several rounds of infection, demonstrating the efficacy of this new system.

## Highlights

• We generated Cre-loxP-regulated fluorescent universal adenoviral vector system.
• The replication of recombinant virus produced by this system is easy to be monitored.
• This system gives higher titer and faster replication of virus in some cases.
• The expression of inserted gene is induced by co-infection of Cre-expression vector.
• We confirmed this system works for the direct reprogramming of MEF into Hepatocyte.

## 1 Introduction

Various virus-based vector systems are used for gene transfer, including adenoviruses, lentiviruses, retroviruses and adeno-associated viruses. Human adenovirus has a non-segmented linear double-stranded DNA genome and 57 serotypes, some of which are pathogenic, such as 3,7, and 21 that induce respiratory disease [9], [13], 8,19 and 37 conjunctivitis [1], 40, 41 and 52 gastroenteritis [6], [8] and the type 14 subfamily severe multi-organ disease [12], [21]. In contrast, Adenovirus type 5 (Ad5) is generally considered to be non-pathogenic and is often used for gene transduction, although a recent report suggests that it induces obesity [22]. Adenovirus can infect both actively dividing and terminally differentiated cells [4] and does not require integration into the host genome for gene expression [23]. Therefore, recombinant adenovirus is useful for gene therapy, especially when transient gene expression is acceptable or beneficial.

pAxCAwtit2 is an adenoviral cosmid vector based on Ad5 [5] which contains the genes required for virus replication but lacks the E1 gene region. The recombinant adenovirus can only proliferate in cells such as HEK293 that express the E1A and E1 B proteins. The E1 gene is replaced by the CAG promoter (fusion of the cytomegalovirus early enhancer, the chicken beta-actin promoter and the splice acceptor of rabbit beta globin) [15], the beta-globin polyadenylation signal and a unique SwaI cloning site, in order to insert the gene to be expressed. Recombinant adenovirus can be produced by transfecting HEK293 cells with this cosmid vector. In general, the percentage of virus producing cells is quite low even if most of the cells are efficiently transfected. From our experience, only 10 to 100 out of 1 × 10^6 cells, or sometimes none of cells produce infectious virus. If virus producing cells are generated, cell death caused by lytic cycles of adenovirus can be observed from 14 to 20 days after transfection. In other words, it takes 2 to 3 weeks to know whether infectious virus will be obtained.

We generated pAxCALRL, which is a Cre-loxP-regulated fluorescent universal vector. The vector has a tag-RFP sequence as a stuffer between two loxP sequences just downstream of a CAG artificial promoter. The newly produced SwaI cloning site for the insertion of genes of interest is situated next to the last loxP sequence. As the RFP gene is expressed instead of the inserted one, the proliferation of the virus can be followed by fluorescence of the RFP positive colonies, which appear from 5 to 7 days after transfection.

Adenovirus can infect a broad range of cells and tissue, however, because of this natural tropism, the use of the virus *in vivo* is limited, due to the risk of toxicity from infection of non-targeted cells [2]. In addition, adenovirus tends to be removed by the immune system, due to pre-existing antibodies [14]. Better adenoviruses for *in-*vivo use are being generated, by modifying tropism and immuno-susceptibility [3], [7], [10], [11], [17], [18]. Recently, it was reported that chemical coupling of adenovirus with a peptide that has a high affinity for a hepatic stellate cell (HSC) surface protein drastically facilitates infection of the corresponding cells [16]. Using this method, Reetz et al. directly reprogrammed hepatic myofibroblasts into hepatocytes *in vivo* by introducing an adenoviral vector that expresses Foxa3, Gata4, Hnf1a, and Hnf4α [20]. This success demonstrates the usefulness of adenovirus *in vivo*, and highlights the need for an easy method to obtain high titers of virus. Our Cre-loxP regulated fluorescent adenoviral expression vector can be expected to produce higher viral titers, as it expresses RFP during viral proliferation instead of the inserted potentially-toxic gene. It is designed to express the inserted gene following infection with an adenovirus that expresses Cre recombinase.

In this article, we described the precise method for generation of Cre-loxP regulated fluorescent adenoviral vectors that express Foxa2 and Hnf4α. Thse adenoviral vectors can be also used for successful direct reprogramming of mouse embryo fibroblasts (MEF) into hepatocyte-like cells [called induced-Hepatocyte (iHep) by Sekiya et al.] [19].

## 2 Materials and methods

### 2.1 Construction of pRSV-LRL

First of all, we prepared the loxP-RFP-loxP construct based on the pRC/RSV (Invitrogen)-based expression vector, pRSV-2A (RDB12341, RIKEN BioResource center). The synthetic oligonucleotides:

loxP-f3 (5′-agcttactagtataacttcgtatagcatacattatacgaagttatcg-3′) and

loxP-r3 (5′-ctagcgataacttcgtataatgtatgctatacgaagttatactagta-3′)

were annealed and cloned into the HindIII and NheI site of pRSV-2A to generate pRSV-loxP. Then, the fragment of the tag-RFP open reading frame PCR-amplified from pTagRFP-N (FP142, Evrogen) that has SpeI and NheI site on the 5′ and 3′ ends, respectively, was inserted into the NheI site of pRSV-loxP to obtain pRSV-loxP-RFP. The SpeI-NheI fragment of pRSV-loxP-RFP was cloned into the SpeI site of pRSV-loxP to complete construction of pRSV-loxP-RFP-loxP(pRSV-LRL).

### 2.2 Construction of the universal adenovirus vector pAxCALRL

In order to clone the loxP-RFP-loxP(LRL) fragment into the pAxCAwtit2 cosmid vector for adenovirus production (RDB05213, RIKEN BioResource Center), the fragment was amplified by PCR using pRSV-LRL as a template and a set of primers, Adlx-5 (5′-ttggtgtgcacctccaagcttact-3′) and Adlx3 (5′-aaatctagaccgcggagctagcgataactt-3′).

#### 2.2.1 Practically;

Step 1. A mixture of Adlx-5 and Adlx-3 were phosphorylated in the following conditions; oligomers 200 pmol each, 5 μl of 10× T4 kinase buffer, 5 μl of 10 mM ATP, 20 U of T4 kinase (M0201S, NEB) in a 50 μl reaction. The reaction was incubated at 37 °C for 60 min and heat-inactivated at 75 °C for 5 min.

Step 2. The loxP-RFP-loxP fragment was amplified by PCR using phosphorylated primers in the following conditions; 1 ng of pRSV-LRL as a template, 2 μl of 10× KOD plus buffer, 0.8 μl of 25 mM MgSO_4_, 2 μl of 2 mM dNTPs 0.2 units of KOD plus (KOD-201, Toyobo) in a 20 μl reaction. The reaction mixture was incubated at 95 °C for 10 min followed by 15 thermal cycles [95 °C for 10 s, 55 °C for 20 s, 72 °C for 40 s]. The PCR product was separated by electrophoresis, excised and electro-eluted using a dialysis bag. The DNA fragment was extracted once with phenol/chloroform and once with chloroform and precipitated with ethanol.

Step 3. 10 μg of pAxCAwtit2 was digested with 40U of SwaI (R0604, NEB) in a 100 μl reaction containing 1 x NEB#3 buffer and 0.1 mg/ml BSA, followed by dephosphorylation by adding 10 μl of 10×CIAP buffer and 20 U of CIAP (M0290, NEB). The reaction was extracted with phenol/chloroform and precipitated with ethanol.

Step 4. For the ligation reaction, the TA-Blunt Ligation Kit (311-06543, Nippon gene) was used. A 5 μl reaction solution was prepared with 50 ng (3.3 fmol) of digested vector from step 3, 0.5 μl of 10 x Enhancer solution, 18 fmol (5 ng) of the insert DNA (prepared in step 2) and 1 μl of 5 x ligation solution. The molecular ratio of vector to insert was approximately 1:5. The reaction solution was incubated at 16 °C for 30 min and used for packaging (Step 5) without further treatment.

Step 5. 5 μl of GigapackIII extract [GigapackIII Plus Packaging Extract (200206, Agilent Technologies)] was added to the 5 μl of ligation reaction from Step 4, and incubated for 2 h at room temperature. The packaged DNA was mixed with 700 μl of *E.coli* DH5α grown to a density of 0.6 OD 600 in LB medium supplemented with 10 mM MgSO_4_. After incubation for 30 min at 37 °C, the bacteria were plated on LB agar supplemented with 1 mg/ml ampicillin. Clones containing the LRL construct in the right orientation were selected by colony-PCR using the specific primers: pAxCAF1(5′-ggcttctggcgtgtgaccggc-3′) and Adlx3 (see above).

### 2.3 Construction of the pAxCALRL-Foxa2 and pAxCALRL-Hnf4α adenoviral vectors

The open reading frames of Foxa2 and Hnf4α were amplified by PCR as described in steps 1 and 2 (see above) using phosphorylated primers:

Foxa2-f: (5′-atgctgggagccgtgaagatggaagggcacgag-3′) and Foxa2-r (5′-ttattaggatgagttcataataggcctg-3′) or

Hnf4-f: (5′-atgcgactctctaaaacccttgcc-3′) and Hnf4-r (5′-ctagatggcttcttgcttggtgatc-3′), respectively.

The PCR products were ligated into the SwaI site of pAxCALRL following the ligation-packaging protocol (see steps 3 to 5, above) except that the primers used for the colony PCR screening were pAxCAF1 and Foxa2-r for Foxa2 and pAxCAF1 and Hnf4-r for Hnf4α.

### 2.4 Production of recombinant adenovirus

The adenoviral cosmid vector in *E.coli* was amplified in 100 ml of LB supplemented with 1 mg/ml ampicillin and purified with the QIAGEN Plasmid Maxi Kit (12163, Qiagen). The vectors, namely pAxCALRL-Foxa2 and pAxCALRL-Hnf4, were linearlized with the Csp45I (BstBI) restriction enzyme (R6571, Promega). HEK293 cells, cultured in DMEM (11885-084, Thermo Fisher Scientific) supplemented with 10% FCS (10099-141, Thermo Fisher Scientific) were transfected with each linearlized cosmid vector using the BBS-mediated calcium phosphate method. 100 μl of solution containing 4 μg of digested pAxCALRL-Foxa2 or pAxCALRL-Hnf4α and 250 mM of CaCl_2_ was prepared and then 100 μl of the 2× BBS solution [50 mM N,N-bis(2-hydroxyethyl)-2-aminoethanesulfonic acid (BES; 391334, Calbiochem), 280 mM NaCl and 1.5 mM Na_2_HPO_4_], was added. After incubation for 20 min at room temperature, the transfection mixture was added to HEK293 cells. The pH of the 2× BBS solution, which is very critical for the efficiency of transfection, was carefully adjusted to between pH6.95 to 6.97 using 1N NaOH. The 2× BBS solution with best transfection efficiency was chosen in pilot transfections. After overnight incubation, the transfected HEK293 cells were spread on 24 well plates and incubated for a further 10–14 days. Typically, RFP positive cells appeared on the day following transfection. The cells began to die from day 7, and were almost all dead after 14 days of transfection, because of the lytic cycles of adenovirus. The adenovirus clone, which came from a single RFP positive colony, was harvested by gathering both medium and cell debris. The virus particles were released from cell debris using 3 freeze-and-thaw cycles. The “1 st virus clones” of AxCALRL-Foxa2 and AxCALRL-Hnf4 adenovirus were stored at −80 °C, until further amplification. In order to amplify the adenovirus for experimental purposes, HEK293 cells in 6 cm were infected with 10 μl of the “1 st virus clones”. The infected cells were incubated until the cells became round and almost detached from the surface of the dish. The cells containing replicated virus were harvested using cell lifter (2008, Corning) together with medium. The medium and the cell debris were subjected to 3 cycles of freeze-and-thaw, and the cell debris was removed by centrifugation. The supernatant was stored at −80 °C as the “2nd virus clones”. Finally, the “3rd virus clones” were prepared by infecting HEK293 cells in 10 cm dish 100ul of 2nd virus clone.

### 2.5 Determination of virus concentration

In order to determine the concentration of virus, Adeno-X Rapid Titer Kit (63225, Clontech) was used following the manufacturer's instruction. Additionally, Adeno-X GoStix (632270, Clontech) was used to check the approximate viral concentration within a short period of time.

### 2.6 Comparison of proliferation rates of adenovirus with and without stuffer

HEK293 cells were cultured overnight in the presence of DMEM supplemented with 0.3% FCS and then infected with AxCALRL-Hnf4 or AxCA-Hnf4. After culturing, the cells were lysed at several time points in TE supplemented with 1% SDS and the DNA was extracted once with phenol and once with chloroform. The relative amounts of viral and cellular DNA were analysed by real-time PCR using specific primers for the adenovirus type 5 hexon (5′-cccgtcaactgagcgcttaatttc-3′ and 5′-acctttaagaaggtggccattacc-3′) and the mouse 28S ribosomal RNA (5′-ggcggccaagcgttcatagc-3′ and 5′-gccaagcacatcaccaaat-3′) genes, respectively. The amounts of viral DNA were normalised with 28S gene DNA. The log10 of viral DNA amounts were plotted against time after infection. The same experiment was done for their Foxa2 counterparts, AxCALRL-Foxa2 and AxCA-Foxa2.

### 2.7 Detection of cre-loxP dependent recombination

MEF was infected with AxCALRL-Foxa2, AxCALRL-Hnf4α and AxCANCre, (RDB01748, RIKEN Bioresource center) following the protocol described above. After 2 to 7 days of infection, the cells were lysed with 1% SDS in TE and the DNA was extracted once with phenol and once with chloroform. Genomic DNA was analysed by PCR using AmpliTaq Gold^®^ 360 Master Mix (4398881, Thermo Fisher) and a set of primers, pAxCAF1 (indicated as “primer 1” in Fig. 2A) and pAxCALRL-r (5′-tctagaccgcggtacctcagctagcg-3′, “primer 2” in Fig. 2A), which was designed to amplify both excised and non-excised viral DNA. The PCR products were subjected to agarose gel electrophoresis to defect the different size products. For quantitative analysis, different sets of primers were used: RFP 5-f (5′-ggtgtttactatgtggaccatagac-3′, “primer 3” in Fig. 2A) and pAxCALRL-r for the non-excised viral genome, and pAxCAF1 and pAxCALRL-r for both the excised and non-excised viral genomes. The reactions, which containing Power SYBR Green PCR Master Mix (4368702, Thermo Fisher) and the indicated primers, were analysed using the StepOnePlus™ Real-Time PCR System (4376600, Thermo Fisher) with following thermal cycle condition; pre-incubation 95 °C for 10 min then 95 °C for 10 s, 60 °C for 30 s and 72 °C for 30 s.

### 2.8 Generation of induced-Hepatocyte (iHep)

Direct reprogramming of mouse embryo fibroblasts (MEF) to Hepatocyte was performed according the method established by Sekiya et al. *(Sekiya and Suzuki)*. 2.5 × 10^4 cells/well of MEF were plated on 12 well dishes and infected with AxCALRL-Foxa2 and AxCALRL-Hnf4α at a MOI (multiplicity of infection) of 10. On the next day, the cells were infected with AxCANCre at a MOI of 20 for Cre-loxP dependent DNA recombination. This infection process was repeated every 7 days up to day 28, giving a total of 5 separate infections. MEF were cultured first in DMEM + 10% FCS for two days, followed by Hepato-medium(-/-) [1:1 mixture of DMEM and F-12 (21331, Life technologies) supplemented with 10% FCS, 1 μg/ml of insulin, 0.1 μM of dexamethasone, 10 mM nicotinamide, 2 mM L-glutamine 50 μM beta-mercaptoethanol and 1× penicilin/Streptomycin]. The medium was changed every two to three days until day 18. From day 18, Hepato-medium(+/ + ) [Hepato-medium(-/-) supplemented with 20 ng/ml of hepatocyte growth factor (HGF, PHG0324,Life technologies) and 20 ng/ml of epidermal growth factor (EGF, E9644, Sigma-Aldrich)]. The medium was changed every two to three days, until days 35 to 40.

### 2.9 Real-time RT-PCR

Total RNA was harvested and extracted using TRIsol (15596-018, Thermo Fisher Scientific) following the manufacturer’s instruction. The total RNA was extracted from iHep or mock(AxCALRL and AxCANCre) infected cells in 12 well plate and 1/20 of the sample were used for reverse transcription using Superscript III reverse transcriptase (18080044, Thermo Fisher Scientific). For real-time PCR, 1/90 of the reverse-transcribed cDNA was used per well, using Power SYBR Green PCR Master Mix and the following set of primers:

MaoA; 5′-gctgtcaaaatcaagtgcatggtgtattac-3′ and 5′-tttccgggcaagtatgaagcccatgat-3′,

GstA4; 5′-gctatgatttgatactgtcaagagcta-3′ and 5′-tcttaaatgcctgcagcagagggaagt-3′,

MaoB; 5′-gagagagcagccagagagattctt-3′ and 5′-gtctctccaggaaggtactggtaa-3′,

Alpha fetoprotein; 5′-attggttacacgaggaaagcacc-3′and 5′-agcagtggctgataccagagtt-3′,

Albumin; 5′-ggcacagtgcttgctgaatttc-3′ and 5′-ctctgatcttcaggaagtgtac-3′,

Cadherin; 5′-tgctcatgtttcccagcgtgta-3′ and 5′-aatggcttctctatccagaggc-3′, and GPDH;5′-ccacttgaagggtggagcca-3′ and 5′-tcatggatgaccttggccag-3′.

Each value was standardized by dividing with that of GPDH and shown as “relative expression level”. The presented data were means of triplicate experiments.

### 2.10 PAS staining

iHep and mock(AxCALRL and AxCANCre) infected cells in 12 well plates were washed with PBS(−) and fixed in with 4% depolymerized paraformaldehyde in PBS for 15 min. After washing with water, the cells were oxidized in 0.5% periodic acid solution (40911, Muto pure chemicals) for 5 min, rinsed in water and placed in cold Schiff's reagent (40931, Muto pure chemicals) for 15 min. The cells were washed three times with sulphite solution (0.5% NaHSO_3_ supplemented with 0.05N HCl, 40941, Muto pure chemicals) and once with water. The cells were counterstained in Mayer's hematoxylin for 2 min and washed in water until the colour was developed.

### 2.11 Immunofluorescence

After PAS staining, the cells were washed and blocked with 5% goat serum (16210-064, Thermo Fisher Scientific) in PBST (PBS- supplemented with 0.1% Tween 20) for 30 min. The cells were incubated with 1/800 dilution of mouse anti-cadherin (610181, BD Biosciences) and rabbit anti-albumin (102419, GeneTex) antibodies in PBST for 1 h. After washing with PBST, the cells were incubated with goat anti-mouse IgG Alexa Flor555 (A21422, Thermo Fisher Scientific) and goat anti-rabbit IgG Alexa Flor 488 (111–545-144, Jackson ImmunoResearch) for 60 min. Nuclei were stained with a 1/10000 dilution of Hoechst 33342(H3570, Thermo Fisher Scientific) in PBS, after incubation with secondary antibody. Finally, the cells were washed with PBS and photographed with a fluorescent microscopy (IX71, Olympus).

## 3 Results

### 3.1 Construction of a cosmid vector for adenovirus production

**Fig. 1:**
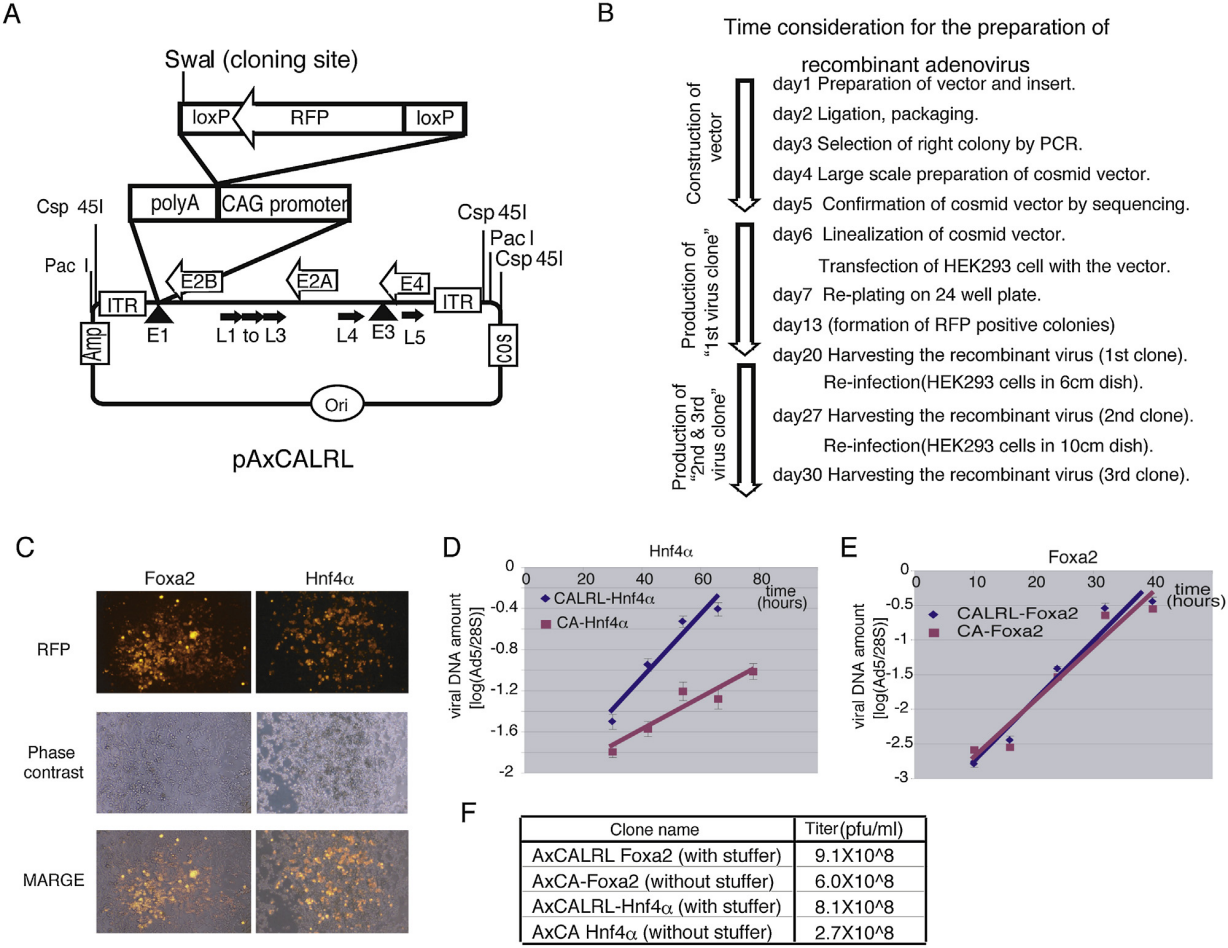
A. Schematic representation of the constructed universal adenoviral vector, pAxCALRL. The SwaI restriction site located next to 2nd loxP sequence is used for insertion of genes of interest. All the genes essential for the adenovirus production except E1, are located between two ITR (Inverted terminal repeat). Amp, Ori and Cos indicate the beta-lactamase gene (ampicillin resistance), the ColE1 origin and Cos sequence, respectively. For production of infectious adenovirus particle by transfection of HEK293 cells, the vector can be linearized by digestion with Csp45I or PacI restriction enzymes. B. Flowchart and timetable for preparation of recombinant adenovirus. C. Microscope images of HEK293 cells producing AxCALRL-Foxa2 or AxCALRL-Hnf4α recombinant adenovirus. HEK293 cell cells were transfected with pAxCALRL-Foxa2 or pAxCALRL-Hnf4α, and re-plated in 24 well plates. Seven days after transfection, RFP positive cells were visualized by fluorescence microscopy. As the budded adenovirus particles tends to infect neighbouring cells, the virus producing cells can be observed as RFP positive colonies. D. and E. Proliferation rates of adenoviruses made to express Hnf4α (D) or Foxa2(E) from vectors that contain (CALRL, blue diamonds) or lack (CA, red squares) stuffers. HEK 293 cells were infected with the indicated recombinant viruses and the total DNA were extracted at several time points after infection. The amounts of viral DNA detected by real-time PCR were normalised to the cellular 28S gene. F. The final titers of adenovirus (the “3rd virus clone” from 10 cm dish).

The pAxCALRL universal cosmid vector was constructed as described in the Materials and Methods (see the schematic structure in Fig. 1A). This vector can be used to efficiently produce recombinant virus within 20–30 days (see the scheme and the time considerations in Fig. 1B). The cloning methods for cosmid vectors are similar to plasmids, but certain precautions are necessary. Cosmid vectors preferentially ligate to short minor DNA fragments. Long PCR-prepared fragments have to be purified by techniques such as agarose gel electrophoresis, to remove even minor amounts of shorter by products. Glass beads-based methods should be avoided for the extraction of DNA from gels, since they can damage DNA, leading to the cloning of deleted fragments. Colony-PCR methods are preferred to select desired clones, since the copy number of cosmid vector DNA in *E.coli* is relatively low, in comparison with plasmid vectors. Point mutations or deletions of the insert are sufficiently frequent to merit verification of the DNA sequence, in contrast to plasmid cloning. The ligation-packaging protocol gave 14 and 33 colonies for Foxa2 and Hnf4α, respectively, and 2 out of 8, and 3 out of 14 clones had the insert in the right orientation, respectively.

Linearized pAxCALRL-Foxa2 and pAxCALRL-Hnf4α were introduced into HEK293 cells in 6 cm dishes and re-plated on 24 well plates the next day. RFP positive cells appeared one day after transfection, and RFP positive colonies, which are a sign of re-infection by adenovirus, appeared after 6 to 7 days (Fig. 1C). Viral clones from wells with single colonies were amplified in HEK293 cells in 6 cm and then 10 cm plates. Finally, we obtained 10 ml of AxCALRL-Foxa2 and AxCALRL-Hnf4α recombinant adenoviruses with titers of 9.1 × 10^8 pfu/ml and 8.1 × 10^8 pfu/ml, respectively. In contrast, the titers of their “non-stuffer” counterparts, prepared in the same way, were 6.0 × 10^8 pfu/ml for AxCA-Foxa2 and 2.7 × 10^8 pfu/ml for AxCA-Hnf4α, which were two thirds and one thirds less than those “with stuffer”, respectively (Fig. 1F).

### 3.2 Comparison of the rates of proliferation of adenoviruses with and without stuffer

In order to compare the effect of stuffer on the efficiency of viral replication, we infected HEK293 cells with AxCALRL-Hnf4α (with stuffer), AxCA-Hnf4α (without stuffer), AxCALRL-Foxa2 (with stuffer) and AxCA-Foxa2 (without stuffer) and extracted DNA at various time points. The amounts of viral and cellular DNA were analysed by real-time PCR and their ratio was used to estimate the amount of viral DNA per cells at each time point. AxCALRL-Hnf4α replicated faster than AxCA-Hnf4α, suggesting that inhibiting Hnf4α expression with stuffer increased the yield of virus (Fig. 1D). In contrast, AxCALRL-Foxa2 and AxCA-Foxa2 increased at the same rate, indicating that the expression of Foxa2 does not affect proliferation of adenovirus (Fig. 1E).

### 3.3 Efficiency of cre-dependent recombination of AxCALRL DNAin MEF

**Fig. 2:**
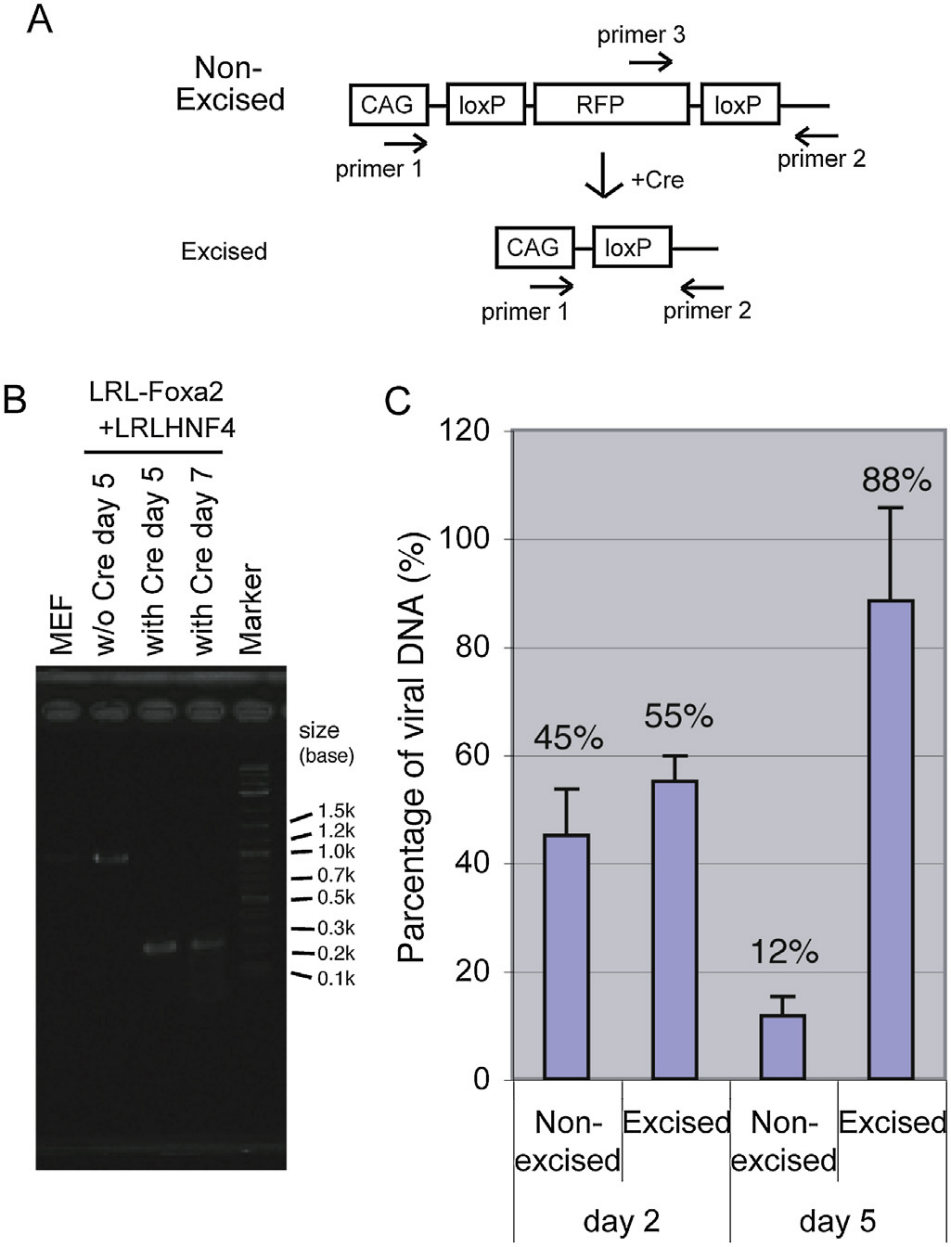
Efficiency of Cre-dependent recombination of AxCALRL viral DNA in infected MEF. A. Schematic structure of AxCALRL around the LoxP sites before and after Cre-dependent recombination. Primer 1(pAxCAF1) and 2(pAxCALRL-r) detect both non-excised and excised DNA, whereas primers 2 and 3 (RFP 5-f) are specific for non-excised DNA. B. Confirmation of Cre-dependent DNA recombination. MEF infected with AxCALRL-Foxa2 or AxCALRL-Hnf4, with or without AxCANCre, were cultured and the DNA was extracted 5 and 7 days after infection. Viral DNA was amplified by PCR using primers 1 and 2. The products were separated by agarose electrophoresis. The 250 bp and 1011 bp DNA fragment correspond to excised and non-excised viral DNA, respectively. C. Percentage of excised and non-excised viral DNA in infected MEF. The MEF infected with AxCALRL-Foxa2, AxCALRL-Hnf4α and AxCANCre were cultured for 2 and 5 days and extracted DNA was analysed by real-time PCR using primers 1 and 2 (excised), and 2 and 3 (non-excised). Note that primers 1 and 2 did not detect non-excised DNA in these experimental condition, as described in the results section.

In order to confirm that Cre recombinase effectively recombined the viral genome, MEF were infected with the adenoviral vectors AxCALRL-Foxa2 and AxCALRL-Hnf4α. One day later, the cells were infected with or without the adenoviral vector that expresses Cre-recombinase. DNA was extracted 2, 5 and 7 days after the first infection, and analysed by PCR using primer 1 (pAxCAF1) and primer 2 (pAxCALRL-r), which were designed to detect both the excised and the non-excised viral genomes (Fig. 2A). With Cre recombinase, the 250 bp excised DNA fragment was detected at days 5 and 7, whereas without Cre the 1011 bp non-excised fragment was detected in the day 5 sample, indicating that the majority of viral DNA was excised within 4 day after the introduction of Cre (Fig. 2B). Non-excised viral DNA was measured with primers 2 and 3 that are specific for non-excised DNA (Fig. 2A). We found by real-time PCR that 55% of viral genome was excised at day 2 and 88% at day 5 (Fig. 2C). In these experimental condition, only the excised DNA was detected with primers 1 plus 2, as shown by electrophoresis of the PCR product, probably due to the short extension time and the preference for amplification of short DNA fragments.

### 3.4 Induction of hepatocytes by direct reprogramming

**Fig. 3:**
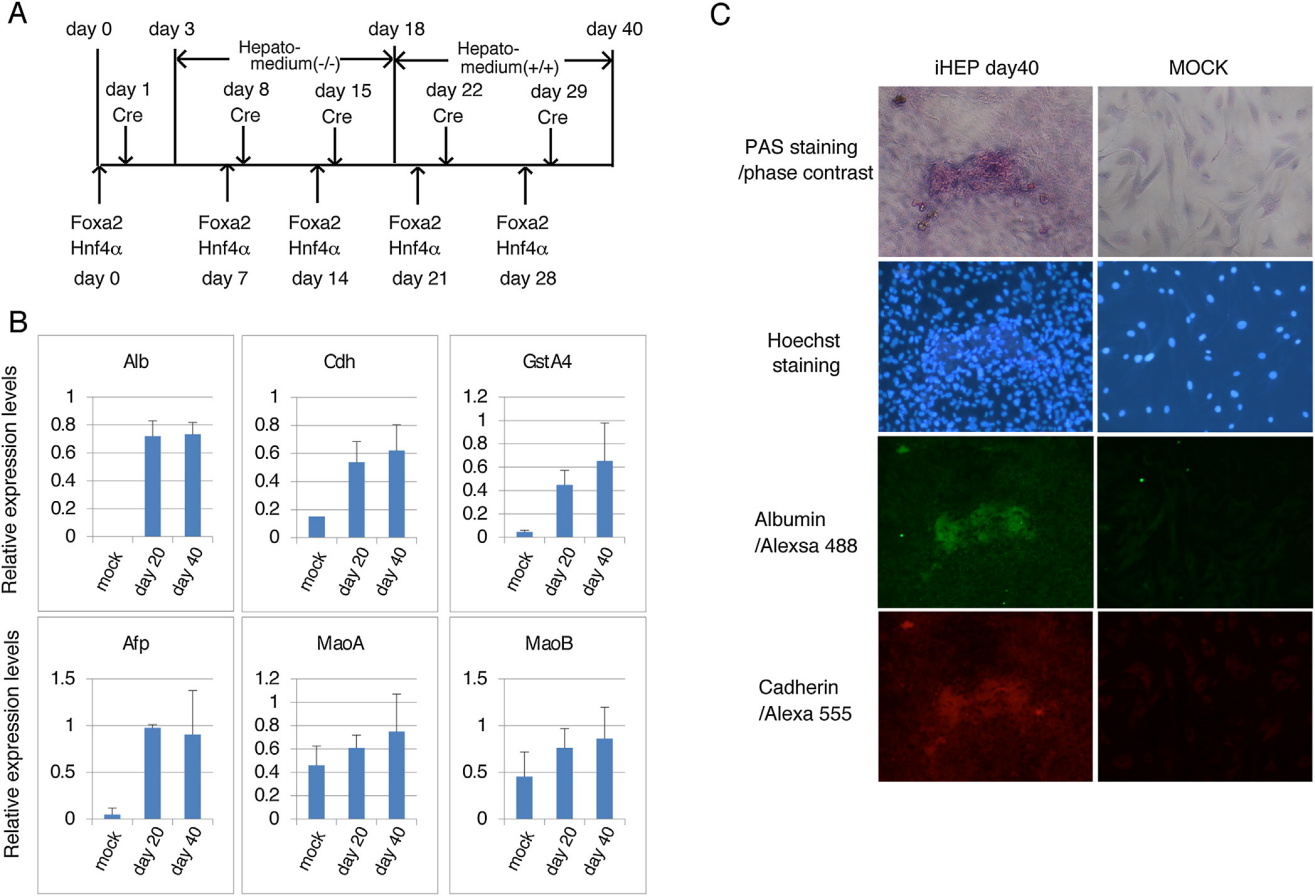
Induction of hepatocyte-like cells (induced-Hepatocytes, iHep) from MEF by the expression of Hnf4α and Foxa2 using recombinant adenovirus. A. Experimental scheme for the induction of hepatocytes. Once a week, cells were infected with AxCALRL-Hnf4α and AxCALRL-Foxa2 followed by AxCANCre on the next day. The cells were cultured in Hepato-medium(-/-) from day 3 to day 18 and in Hepato-medium(+/ + ) from day 18 to day 40. B. Expression of hepatocyte marker genes. The total RNA were extracted at day 20 and day 40 and analysed by real-time RT-PCR using specific primers for Albumin (Alb), Cadherin (Cdh), GstA4(Glutathione S-transferase A4), AFP (Alpha fetoprotein), MaoA and MaoB (Monoamine oxidases A and B, respectively). The expression levels of mRNA were normalised by that of GPDH. C. Microscope images of induced-Hepatocytes. The cells were fixed with formaldehyde 40 days after the 1 st infection and subjected to Periodic acid-Schiff staining followed by immunostaining using rabbit anti-albumin and mouse anti-cadherin as 1 st antibodies and anti-rabbit and anti-mouse IgG antibodies conjugated with Alexa Fluore 488 and 555, respectively, as 2nd antibodies. Nuclear DNA were stained with Hoechst 33342.

In order to assess whether these Cre-loxP regulated fluorescent adenoviral expression vectors could be used for direct reprogramming of hepatocytes, MEF were infected with recombinant adenovirus that express Foxa2 (AxCALRL-Foxa2), Hnf4α (AxCALRL-Hnf4α) followed by the Cre expression virus, AxCANCre. After culturing for 20 and 40 days with the indicated protocol (Fig. 3A), Real-time RT-PCR was used to measure the expression of mRNAs for hepatocyte differentiation markers [Cadherin, Albumin, GstA4 (Glutathione S-transferase A4), AFP (Alpha fetoprotein), MaoA and MaoB (monoamine oxidase A and B)]. The expression of mRNAs for Cadherin, Albumin, GstA4 and AFP were significantly increased 20 and 40 days after the first infection and those for MaoA and MaoB were augmented to some extent (Fig. 3B). As hepatocyte cells are rich in glycogen, PAS staining was performed on cells 40 days after infection. Some colonies stained positively with PAS. Samples stained with PAS were used for immunofluorescent staining to detect the hepatocyte marker proteins, Cadherin and Albumin. The PAS-positive cells expressed Cadherin (green fluorescent cells) and Albumin (red fluorescent cells). These results show that induced hepatocyte can be generated by the expression of Foxa2 and Hnf4α using adenoviral vectors.

## 4 Discussion

Cosmid vectors are not as commonly used as plasmid-based vectors, but they can be powerful tools for the preparation of adenovirus vectors. They can hold more than 40 kb of DNA, which is enough to carry the whole adenovirus genome. The vector needs to be digested with the restriction enzyme Csp45I before the introduction into HEK293 cells, in order to produce infectious virus. Linearisation next to replication origin of adenovirus (the ITR; inverted terminal repeat) is thought to favour binding of the precursor terminal protein (pTP) complex, that contains viral DNA polymerase and deoxycytidine needed for DNA replication [5]. Cellular DNA repair mechanisms are thought to generate single stranded DNA at the site of linearisation, which favours binding of the complex. The low efficiency of this process may limit virus production. In our experiments, we transfected semi-confluent HEK293 cells in 6 cm dishes and re-plated them in 24 well plates. RFP positive colonies appeared in most of the wells, but the number of the colonies was 1 to 3 per well; therefore only 30 to 100 virus-producing cells were obtained with one transfection. In our experience, this efficacy is common and is considered to be a successful preparation of recombinant adenovirus. RFP positive cells do not always lead to the formation of colonies, probably because they do not produce infectious virus. AxCALRL contains stuffer DNA, which prevents expression of potentially toxic inserted-gene products. AxCALRL-Hnf4α has a doubling time two hold faster than AxCA-Hnf4α, indicating that Hnf4α expression inhibits viral replication. In contrast, Foxa2 expression has little effect on doubling time. The final amount of virus obtained with AxCALRL-Foxa2 (with stuffer) from 10 cm dishes is 1.5 fold higher than with AxCA-Foxa2, suggesting that high concentrations of Foxa2 in the cell might inhibit virus production to some extent. These results indicate that vectors with stuffers can increase the yield of virus. From our experience, recombinant adenoviruses with gene inserts up to 4 kb without stuffer take longer to replicate, and hinder obtaining sufficient virus for further manipulation. In this case, we recommend using pAxCALRL or equivalent vector systems with stuffers.

Control experiments, for the efficiency of Cre-loxP dependent DNA recombination in targeted MEF cells, demonstrated that 50% of the viral genomes were excised within one day and 90% within 4 days after infection of the recombinant adenovirus that expresses Cre DNA recombinase (AxCA-NCre). 5 days after the 1 st infection we could still observe RFP expression by fluorescent microscopy, as expected from the 10% that was not excised. This residual expression of RFP can even be helpful, to confirm infection of targeted cells in some experiments. Otherwise, infecting 10 times more AxCA-NCre might be necessary to increase DNA recombination and reduce RFP expression.

Several round of infection were required for reprogramming of MEF into hepatocytes. Single infections were not sufficient to induce expression of hepatocyte specific markers. This could be a consequence of the loss of non-integrated adenovirus from the actively dividing cells. We infected once a week, to maintain expression of the reprogramming factors Hnf4α and Foxa2. With these conditions, we observed increased expression of hepatocyte marker genes, including Cadherin, Albumin, GstA4, AFP, MaoA and MaoB, as well as accumulation of glycogen, a characteristic of hepatocytes. Cells with accumulated glycogen also expressed Cadherin and Albumin. In conclusion, we successfully generated induced-hepatocyte by direct reprogramming with our Cre-loxP regulated fluorescent adenoviral expression vector system. With this vector, it is easy to monitor viral replication by fluorescence and obtain higher titers of virus, in comparison with other systems. Therefore, this recombinant viral expression system should be useful for many other applications.

## Additional information

The complete sequence of pAxCALRL and its GFP counterpart, pAxCALGL were presented in Genbank as accession numbers KX529075 and KX589554, respectively. All the materials with RDB number can be destributed from RIKEN Bioresource Center DNA bank (http://dna.brc.riken.jp/index.html). The rest of adenovirus-related materials will be available by contacting K.N. or dnabank@brc.riken.jp.

## Conflict of interest statement

The authors have declared no conflicts of interest.
